# Pain‐relieving effectiveness, quality of life and tolerability of repeated capsaicin 8% patch treatment of peripheral neuropathic pain in Scandinavian clinical practice

**DOI:** 10.1002/ejp.1180

**Published:** 2018-02-01

**Authors:** P. Hansson, T.S. Jensen, G. Kvarstein, M. Strömberg

**Affiliations:** ^1^ Department of Molecular Medicine & Surgery Karolinska Institutet Stockholm Sweden; ^2^ Department of Pain Research & Treatment Division of Emergencies and Critical Care Oslo University Hospital Norway; ^3^ Department of Neurology and Danish Pain Research Center Aarhus University Hospital Denmark; ^4^ UIT The Arctic University of Norway Tromsø Norway; ^5^ Astellas Pharma A/S Nordic Operations Kastrup Denmark

## Abstract

**Context:**

Clinical trials have demonstrated the efficacy and safety of the capsaicin 8% patch in patients with peripheral neuropathic pain (PNP); however, few studies have assessed this treatment in a clinical practice.

**Objective:**

To determine whether treatment and re‐treatment with the capsaicin 8% patch reduce PNP intensity in clinical practice.

**Methods:**

Three non‐interventional, observational studies were concurrently conducted in Denmark, Norway and Sweden. Patients with probable or definite PNP received one or two treatments with the capsaicin 8% patch according to usual clinical practice. All analyses were performed on combined data.

**Results:**

Overall, 382 and 181 patients received treatment and re‐treatment, respectively, with the capsaicin 8% patch. At the group level, a significant reduction in mean level of ‘usual pain’ intensity (Numerical Pain Rating Scale) over the last 24 h’ score was observed from baseline to Weeks 2 through 8 [−1.05 (95% confidence interval: −1.27, 0.82); *p* < 0.001] with 28% and 31% of patients reporting a ≥30% reduction in pain after first treatment and re‐treatment, respectively. Improvements in health‐related quality of life (EQ‐5D‐3L index) and overall health status (Patient Global Impression of Change) were observed early (Week 1) and throughout the treatment periods. Most application site reactions subsided within a week after treatment. Following treatment and re‐treatment, 57% and 71% of patients, respectively, were willing to undergo further treatment with the capsaicin 8% patch.

**Conclusion:**

In Scandinavian clinical practice, capsaicin 8% patch treatment was associated with significant reductions in pain intensity and was well tolerated with over half of patients willing to undergo re‐treatment.

## 1 Introduction

Neuropathic pain is defined as pain due to a lesion or disease of the somatosensory nervous system [International Association for the Study of Pain (IASP), 2012]. Peripheral neuropathic pain may manifest itself in a number of aetiologies, including traumatic nerve injury, radiculopathy, polyneuropathy and after herpes zoster (Finnerup et al., 2015). Commonly used pharmacological treatments include antidepressants, anticonvulsants, topical lidocaine and opioids. These treatments, however, are limited by central nervous system adverse effects, such as somnolence and dizziness, and for the latter, the potential for drug dependence (Attal et al., 2010; Finnerup et al., 2010, 2015; Tesfaye et al., 2011; Smith et al., 2012; Gahr et al., 2013). In addition, current evidence indicates that less than half of patients with neuropathic pain obtain sufficient pain relief with current systemic analgesics (Finnerup et al., 2010).

Capsaicin is a highly selective agonist of the TRPV1 receptor of the A delta and C fibres. Activation of TRPV1‐expressing nociceptors leads to defunctionalization of TRPV1‐containing sensory axons, and reversible retraction of epidermal and dermal nerve fibres, followed by inhibition of excitability after a single treatment (Szallasi and Blumberg, 1999; Kennedy et al., 2010; Anand and Bley, 2011). The capsaicin 8% patch is designed to rapidly deliver a high concentration of capsaicin to epidermal nerve endings and is indicated for the treatment of peripheral neuropathic pain (PNP) in adults either alone or in combination with other medicinal products for pain (European Medicines Agency, 2015). The clinical efficacy and safety of this patch have been documented in a comprehensive phase II and III clinical development programme, showing sustained and clinically relevant pain relief after a single application (Backonja et al., 2008; Simpson et al., 2008, 2016; Irving et al., 2011; Clifford et al., 2012; Vinik et al., 2016). A meta‐analysis of seven randomized controlled clinical studies of single application of capsaicin 8% patch for the treatment of PHN and HIV‐associated PNP showed that almost half of the patients achieved a ≥30% reduction in pain intensity within 12 weeks, which was significantly superior to controls (Mou et al., 2014). A recent study showed that the number needed to treat to achieve a 50% reduction in pain was 10.6 (Finnerup et al., 2015). The outcomes of single applications of the capsaicin 8% patch for the treatment of PNP have been documented in clinical practice (Maihofner and Heskamp, 2013, 2014; Haanpaa et al., 2016), and recently, repeat applications were found to be effective and well tolerated in a 52‐week real‐world European study (Mankowski et al., 2017).

The main aim of this combined analysis of three non‐interventional, observational studies, performed in Norway, Sweden and Denmark, was to investigate patient's PNP intensity following treatment and re‐treatment with the capsaicin 8% patch in Scandinavian clinical practice. Here we report the effectiveness, tolerability, patient‐reported health‐related quality of life (HRQoL), concomitant use of medications due to PNP and the willingness to receive additional treatment(s) following treatment and re‐treatment with the capsaicin 8% patch.

## 2 Materials and methods

### 2.1 Study design and participants

Three prospective, non‐interventional, observational studies with identical protocols were concurrently conducted between November 2010 and September 2012 in Denmark, Norway and Sweden in accordance with the principles of the Declaration of Helsinki, International Conference on Harmonization guidelines and local ethical and legal requirements.

Patients were eligible for inclusion if they were ≥18 years old; had probable or definite PNP in accordance with Treede et al., 2008 (Treede et al., 2008); and had provided written informed consent. Patients diagnosed with PNP due to partial nerve damage, and patients with amputations suffering from residual limb pain, suggesting active afferent nerves, were allowed to enter the studies. Patients with phantom limb pain (total deafferentation pain) only were not included, as this pain is not considered responsive to local capsaicin treatment. Exclusion criteria included: previous treatment with the capsaicin 8% patch; PNP due to total deafferentation; unlikely or possible PNP (Treede et al., 2008); facial pain; diabetic PNP; or unsuitability for treatment with the capsaicin 8% patch (based on the discretion of the treating physician).

### 2.2 Treatment

The capsaicin 8% patch [QUTENZA™ cutaneous patch (capsaicin 179 cutaneous patch (capsaicin 179 mg, 8% w/w), supplied by Astellas Pharma Europe B.V., Leiden, The Netherlands] was applied, as directed in the summary of product characteristics (European Medicines Agency, 2015). At each treatment visit, the size of the application area was assessed, and the severity of the application site reaction and pain intensity was recorded. A maximum of four patches were allowed per treatment. The patch application time was 30 min on the feet and 60 min on other parts of the body (Simpson et al., 2008). After treatment, there was an option of up to six additional follow‐up contacts with the clinic as deemed necessary by the investigator and/or patient (Table 1). No pre‐treatment assessments were carried out prior to re‐treatment. Re‐treatment was offered at the physician's discretion, at the recommended interval of ≥90 days after the previous treatment, consistent with the summary of product characteristics (European Medicines Agency, 2015). Patients were followed for up to 3 months after each treatment.

**Table 1:** Clinic visits/contacts and assessments

	First treatment baseline	Re‐treatment baseline	Week 1 Visit 1	Week 1 Visit 2	Week 2	Week 4	Week 8	Week 12
Days from patch application^a^			3 ± 3	3 ± 3	14 ± 7	30 + 15/−8	60 ± 14	90 + 30/−15
Assessment								
NPRS ‘pain now’^b^	✓		✓	✓	✓	✓	✓	✓
NPRS ‘usual’ pain^c^	✓		✓		✓	✓	✓	✓
EQ‐5D‐3L	✓		✓		✓	✓	✓	✓
PGIC			✓		✓	✓	✓	✓
Concomitant medication	✓		✓		✓	✓	✓	✓
Size of application area and patch	✓	✓						
Severity of application site reaction	✓	✓	✓	✓				
Size of painful area^d^			✓		✓	✓	✓	✓

EQ‐5D‐3L, EuroQol five dimensions 3 level questionnaire NPRS, Numerical Pain Rating Scale; PGIC, Patient Global Impression of Change.

a The timing of the follow‐up contacts was determined by the established practice of each centre and patient requirements.

b Pain intensity due to patch application.

c Pain over the last 24 h.

d The baseline size of the pain area was the size of the patch applied at the first visit of each treatment.

### 2.3 Assessments

#### 2.3.1 Effectiveness and tolerability

Patients were assessed by their treating physician or a study nurse. The Numerical Pain Rating Scale (NPRS) [ranging from 0 (no pain) to 10 (worst imaginable pain)] (Farrar et al., 2001) was used to assess ‘usual level pain score for the past 24 h’ (herein referred to as NPRS ‘usual’ pain), pain intensity ‘right now’ and the ‘lowest’ and ‘highest’ level of pain intensity over the last 24 h. HRQoL was assessed by the EuroQol five dimensions 3 level questionnaire (EQ‐5D‐3L) (Rabin and de Charro, 2001). The Patient Global Impression of Change (PGIC) questionnaire was used to measure changes in patients’ overall health status compared to before treatment (Hurst and Bolton, 2004).

The primary effectiveness endpoint was the mean change in pain intensity, evaluated by mean NPRS ‘usual’ pain, from first treatment baseline to the mean average of all values observed between Weeks 2 and 8. The change in the mean NPRS ‘usual’ pain score from baseline (before patch application at treatment visit) to Weeks 2 through 8 was also assessed following re‐treatment. Data from the closing visit in the first treatment period (Week 12) were used as baseline for the re‐treatment period (herein referred to as re‐treatment baseline). At the final follow‐up contact of the first and second treatment periods, patients were asked whether they would be willing to undergo re‐treatment with the capsaicin 8% patch. Other secondary effectiveness endpoints for first treatment and re‐treatment included the proportion of patients with a ≥30%, ≥50% or ≥2 units reduction in mean NPRS ‘usual’ pain score from first treatment baseline and re‐treatment baseline to Weeks 2 through 8 and to Weeks 2 through 12, respectively. Additional secondary endpoints were: time to re‐treatment; change in overall health status using the PGIC questionnaire at each assessment; change in the EQ‐5D‐3L health score from first treatment baseline and re‐treatment baseline to each assessment; change in the size of the painful area (decreased, increased, unchanged) from first treatment baseline and re‐treatment baseline to each assessment; change in the use of concomitant medications due to PNP from baseline to each assessment; willingness to undergo re‐treatment; tolerability (application site reactions; treatment‐related effects [NPRS pain ‘right now’] from first treatment baseline and re‐treatment baseline to each assessment; use of rescue medications).

Treatment‐related effects were recorded as mild (no impact on the patient), moderate (has impact on the patient, but tolerable) or severe (has impact on the patient's daily living and the patient received treatment against the application site reaction). Adverse events were reported by centres in accordance with routine practice for spontaneous adverse event reporting. Due to the non‐interventional nature of the three studies, intense patient follow‐up was not possible.

### 2.4 Statistical methods

Statistical power was not determined due to the non‐interventional nature of the studies. The study protocol estimated that a total of approximately 400 patients (200 in Sweden and 100 each in Denmark and Norway, respectively) would be included in the three studies. As the three studies shared a common design and were performed concurrently, patients likely had similar baseline demographic characteristics, and the complementary effectiveness data from the individual studies provided sufficient justification for pooling study data. The data presented are the post hoc, pooled results from these three studies.

Descriptive statistics were used for presenting age, number of concomitant neuropathic pain medications, NPRS pain levels, size of the treatment area at baseline and tolerability. Time to re‐treatment was calculated using the Kaplan–Meier method.

Numerical Pain Rating Scale scores observed during the first 13 days following first treatment were not included in the primary analysis due to the potential bias from anaesthesia and analgesia pre‐treatment and possible use of rescue medication. Analysis of covariance (ANCOVA) was used for the primary analysis to test for influential baseline characteristics (including sex, age, certainty of PNP diagnosis, PNP aetiology and NPRS pain) and for country, using a significance level of *p *≤* *0.05. For responder endpoints (≥30%, ≥50% or ≥2 units reduction in mean NPRS ‘usual’ pain), logistic regression was also used to determine influential baseline covariates.

## 3 Results

### 3.1 Patient population

A total of 412 patients were enrolled in three concurrent multicenter studies in Sweden (*n = *211, 27 centres), Denmark (*n = *101, 12 centres, including one in Iceland) and Norway (*n = *100, 14 centres). During the first treatment period, 30 patients discontinued [due to ‘lost to follow‐up’ (*n = *11), lack of pain relief (*n = *7), withdrawn consent (*n* = 4), progression of concurrent diagnoses (*n* = 2), surgery/injury of treated area (*n* = 2), incorrect enrolment (*n* = 1), death due to cancer (*n* = 1), sensitivity to capsaicin 8% patch (*n* = 1), hospitalization due to pneumonia (*n* = 1)], leaving 382 patients for primary analysis. Re‐treatment was initiated in 184 patients, but three patients discontinued, leaving 181 patients for analysis (Table S1). The population that completed the first treatment period had a mean age of 53.1 years, 41% of patients were male, and the most common PNP diagnosis was partial peripheral nerve injury (70%) (Table 2). Similar data were observed in the population that completed the second treatment period.

**Table 2:** Demographic data and baseline characteristics of patients that completed treatment

	First treatment (*n = *382)	Re‐treatment (*n = *181)
Gender, *n* (%)		
Male	156 (41)	77 (43)
Female	226 (59)	104 (58)
Age, years		
Mean (SD)	53.1 (16)	51.4 (15)
Median (min–max)	53 (18–88)	52 (18–85)
Certainty of PNP diagnosis, *n* (%)		
Probable	127 (33)	62 (34)
Definite	255 (67)	119 (66)
PNP aetiology, *n* (%)		
Partial peripheral nerve injury	266 (70)	125 (69)
Post‐herpes zoster	51 (13)	22 (12)
Polyneuropathy	19 (5)	6 (3.3)
Other	46 (12)	28 (16)
Concomitant PNP medication, *n* (%)		
Yes	205 (54)	94 (52)
No	177 (46)	87 (48)
Number of concomitant PNP medications (SD)		
Mean (SD)	0.62 (0.78)	0.64 (0.80)
Median (min–max)	0.0 (0–4)	0.0 (0–4)
Size of treatment area, cm^2^		
Mean (SD)	229.6 (195.9)	206.3 (178.7)
Median (min–max)	180 (3–1120)	160 (4–1000)

PNP, peripheral neuropathic pain; SD, standard deviation.

### 3.2 Pain intensity

**Figure 1:**
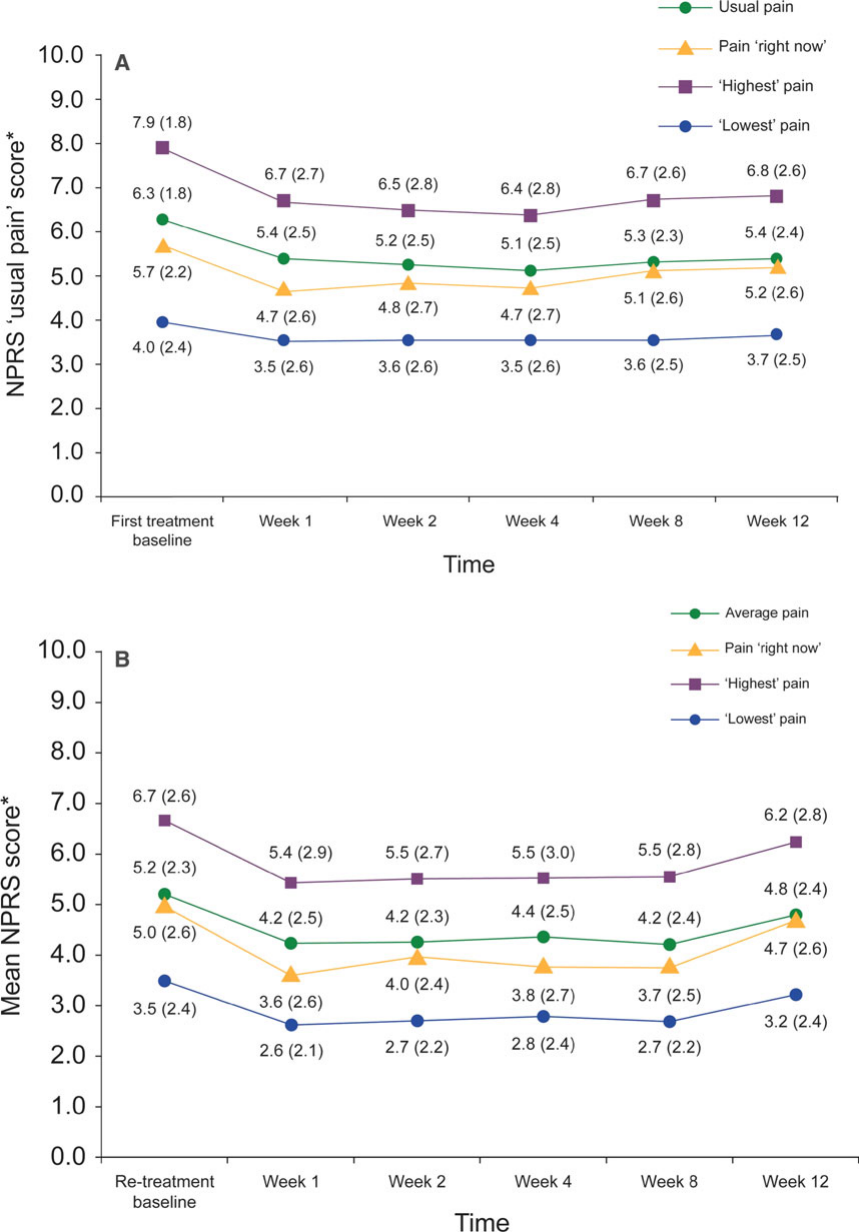
Change in mean pain intensity measured by Numerical Pain Rating Scale (NPRS) after (A) first treatment and (B) re‐treatment with capsaicin 8% patch. NPRS was assessed for ‘usual level of pain for the past 24*** ***h’, pain intensity ‘right now’ and the ‘highest’ and ‘lowest’ pain scores. *Mean (SD).

Following first treatment, the overall group mean NPRS ‘usual’ pain score was significantly reduced from baseline to Weeks 2 through 8 (−1.05; 95% confidence interval: −1.27, −0.82; *p *<* *0.001) (Table 3). A total of 28% (*n = *102) and 17% (*n = *61) of patients had a ≥30% and ≥50% reduction in mean NPRS ‘usual’ pain score, respectively, from first treatment baseline to Weeks 2 through 8, and 33% (*n = *118) had a reduction of ≥2 units (Table 4). Over the 12 weeks, the overall group mean NPRS ‘usual’ pain score decreased from 6.27 [standard deviation (SD) 1.80, *n = *382] at first treatment baseline to 5.39 (SD 2.4, *n = *368) (Fig. 1A).

**Table 3:** Change in the mean NPRS ‘usual’ score for the past 24 h from baseline.^a^

	Weeks 2 through 8	Weeks 2 through 12
First treatment	(*n = *361)	(*n = *381)
Mean (SD)	−1.05 (2.91)	−0.97 (2.04)
95% CI	−1.27, −0.82	−1.18, −0.77
*p* value	˂0.001	˂0.001
Re‐treatment	(*n = *151)	(*n = *169)
Mean (SD)	−0.75 (2.00)	−0.54 (1.87)
95% CI	−1.07, −0.42	−0.83, −0.26
*p* value	˂0.001	˂0.001

CI, confidence interval; NPRS, Numerical Pain Rating Scale; SD, standard deviation.

A total of 382 and 181 patients received first treatment and re‐treatment, respectively.

a Baseline for re‐treatment was the Week 12 assessment from first treatment.

**Table 4:** Responders after each capsaicin 8% patch treatment

	Reduction in NPRS ‘usual’ pain		
	≥30%	≥50%	≥2 units
First treatment, *n* (% [95% CI])			
Baseline to Weeks 2 through 8 (*n = *361)	102 (28 [24, 33])	61 (17 [13, 21])	118 (32 [28, 38])
Baseline to Weeks 2 through 12 (*n = *381)	109 (29 [24, 33])	62 (16 [13, 20])	112 (29 [25, 34])
Re‐treatment, *n* (% [95% CI])			
Baseline to Weeks 2 through 8 (*n = *151)	47 (31 [24, 39])	27 (18 [13, 25])	39 (26 [20, 33])
Baseline to Weeks 2 through 12 (*n = *169)	45 (27 [21, 34])	20 (12 [8, 18])	28 (17 [12, 23])

CI, confidence interval; NPRS ‘usual’ pain, Numeric Pain Rating Scale ‘usual pain in the last 24 h’.

After re‐treatment, there was a significant reduction in the overall group mean NPRS ‘usual’ pain from re‐treatment baseline to Weeks 2 through 8 (−0.75; 95% CI: −1.07, −0.42; *p *<* *0.001). The proportion of patients classified as ≥30% and ≥50% responders after re‐treatment [31% (*n* = 47) and 18% (*n* = 27), respectively] was similar to the observed values after first treatment, and 26% (*n* = 39) of patients had a reduction of ≥2 units. The overall group mean NPRS ‘usual’ pain score decreased from 5.18 (SD 2.32, *n = *177) at re‐treatment baseline to 4.78 (SD 2.40, *n = *162) over the 12 weeks (Fig. 1B). The proportion of ≥30% and ≥50% responders was similar from first treatment baseline and re‐treatment baseline to Weeks 2 through 12 (Table 4).

There were also reductions in the overall group mean NPRS ‘highest’ and ‘lowest’ scores following first treatment and re‐treatment (Fig. 1A and B). A reduction in mean NPRS ‘usual’ pain from first treatment baseline and re‐treatment baseline to Weeks 2 through 8 was reported in each PNP aetiology group (Table 5). Overall, there were no significant differences in pain intensity reported between countries.

**Table 5:** Change in mean NPRS ‘usual’ pain score, from baseline^a^ to Weeks 2 and 8, according to PNP aetiology

Mean change in NPRS ‘usual’ pain (95% CI)	Partial peripheral nerve injury (*n = *266^b^)	Post‐herpes zoster (*n = *51^b^)	Polyneuropathy (*n = *19^b^)	Other (*n = *28^b^)
First treatment	−0.98 (−1.23, −0.71)	−1.03 (−1.59, −0.47)	−0.57 (−1.50, 0.35)	−1.66 (−2.26, −1.05)
Re‐treatment	−0.73 (−1.08, −0.38)	−0.62 (−1.50, 0.25)	−0.44 (−1.91, 1.03)	−0.98 (−1.73, −0.23)

CI, confidence interval; NPRS, Numerical Pain Rating Scale; PNP, peripheral neuropathic pain.

a Baseline for re‐treatment was the Week 12 assessment from first treatment.

b Number of patients in each aetiology group at baseline.

### 3.3 Patient global impression of change

Approximately half the patients reported an improvement in overall health status at Week 12 after first treatment; 21% (*n = *80) reported either much improvement or very much improvement (Table 6). During the re‐treatment period, 66% (*n = *111) of the patients reported an improvement and 44% (*n = *74) reported much improved or very much improved. A worsening of health status or no change was reported by 12% (*n* = 43) and 42% (*n* = 156) of patients after first treatment, respectively, decreasing to 7% (*n* = 12) and 27% (*n* = 45) after re‐treatment.

**Table 6:** Summary of the answers to the Patient Global Impression of Change after first treatment and re‐treatment with capsaicin 8% patch

	Week 1	Week 2	Week 4	Week 8	Week 12
First treatment, *n* (%)	(*n = *267)	(*n = *278)	(*n = *266)	(*n = *215)	(*n = *374)
I feel very much worse	6 (2)	2 (1)	4 (2)	2 (1)	4 (1)
I feel much worse	6 (2)	7 (3)	9 (3)	6 (3)	6 (2)
I feel slightly worse	25 (9)	25 (9)	18 (7)	21 (10)	33 (9)
I feel no change	86 (32)	99 (36)	83 (31)	82 (38)	156 (42)
I feel slightly improved	89 (33)	85 (31)	85 (32)	51 (24)	95 (25)
I feel much improved	28 (11)	39 (14)	36 (14)	30 (14)	48 (13)
I feel very much improved	27 (10)	21 (76)	31 (12)	23 (11)	32 (9)
Re‐treatment, *n* (%)	(*n = *131)	(*n = *108)	(*n = *103)	(*n = *82)	(*n = *169)
I feel very much worse	1 (1)	0	0	0	0
I feel much worse	3 (2)	1 (1)	3 (3)	0	3 (2)
I feel slightly worse	6 (5)	3 (3)	3 (3)	1 (1)	9 (5)
I feel no change	19 (15)	12 (11)	23 (22)	17 (21)	45 (27)
I feel slightly improved	43 (33)	40 (37)	29 (28)	23 (28)	37 (22)
I feel much improved	27 (21)	28 (26)	21 (20)	24 (29)	42 (25)
I feel very much improved	32 (24)	24 (22)	24 (23)	17 (21)	32 (19)

### 3.4 EQ‐5D‐3L

**Figure 2:**
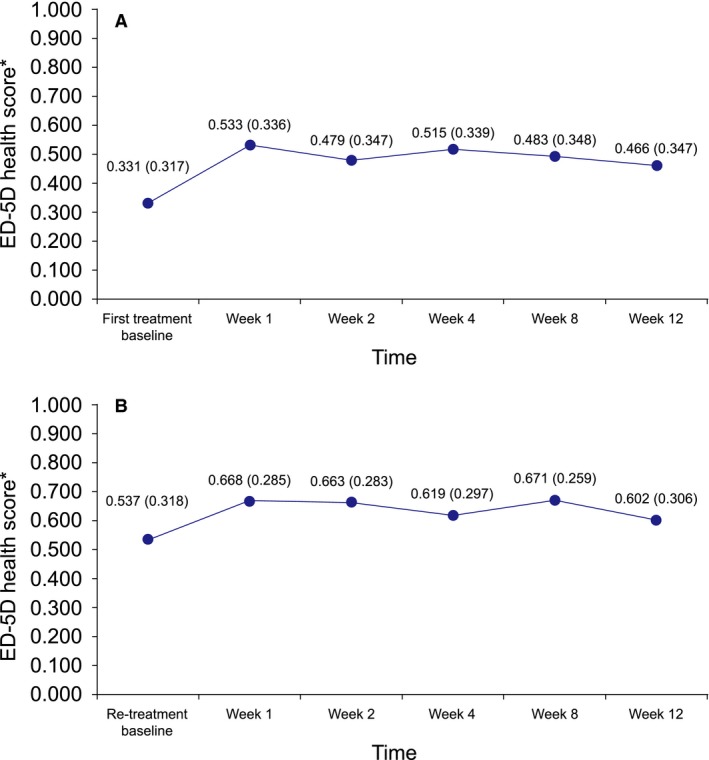
Mean values for EQ‐5D‐3L health scores by visit after the first treatment (A) and re‐treatment (B). *Mean (SD).

At first treatment and re‐treatment baseline, the dimension with the lowest scores was pain/discomfort with 58% (*n *=* *222) and 30% (*n *=* *50) of patients, respectively, reporting extreme problems. The change in the overall group mean (SD) EQ‐5D‐3L health score from first treatment baseline [0.331 (0.317)] to Week 2 was 0.148 utils, twofold the minimally important difference of 0.074 utils (Walters and Brazier, 2005) (Fig. 2A), with 55% (*n *=* *205) of patients experiencing an improvement. At Week 2 following re‐treatment, the health score increased by 0.126 utils with 40% (*n *=* *65) experiencing an improvement. Overall, the improvements in the mean EQ‐5D‐3L health scores from first treatment baseline and re‐treatment baseline to Week 12 were 0.135 utils and 0.065 utils, respectively (Fig. 2A and B), with 52% (*n *=* *192) and 32% (*n *=* *52) of patients experiencing an improvement.

### 3.5 Willingness to undergo re‐treatment

Of the 382 patients who completed the first treatment period, 216 patients (57%) were willing to receive a second treatment (Table 7). The primary reason for treatment discontinuation was unsatisfactory pain relief (73%; *n* = 137). Re‐treatment was initiated for 194 patients (51%), with 181 patients (47%) completing the re‐treatment period. In 55% (*n = *100) of patients who received re‐treatment, patch application was performed directly after the first treatment period (on the final assessment day). After re‐treatment, 128 patients (71%) were willing to receive an additional (third) treatment. The median time between first treatment and re‐treatment was 100 (range 43–289) days.

**Table 7:** Willingness to continue treatment with capsaicin 8% patch

*n* (%)	First treatment (*n = *382)	Re‐treatment (*n = *181)
Patient agrees to re‐treatment		
No, absolutely not		
Unsure	40 (10)	24 (13)
Yes, definitely	216 (57)	128 (71)
Patient's reason no or unsure about re‐treatment		
Not relevant, no longer have pain due to PNP	6 (2)	4 (2)
Unsatisfactory pain relief	132 (35)	38 (21)
Initial adverse event, pain due to patch	12 (3)	1 (1)
Other reason	14 (4)	8 (4)
Patient will receive re‐treatment		
No	187 (49)	55 (30)
Yes	194 (51)	126 (70)
Physician's reason for no or unsure about re‐treatment		
Patient not interested in re‐treatment	16 (4)	3 (2)
Not relevant, no longer have pain due to PNP	15 (4)	5 (3)
Unsatisfactory pain relief	137 (36)	39 (22)
Initial adverse event, application site reaction due to patch	1 (˂0.5%)	0
Initial adverse event, pain due to patch	4 (1)	1 (1)
Other reason	14 (4)	7 (4)

PNP, peripheral neuropathic pain.

### 3.6 Change in size of painful area

At Week 2, 34% (*n = *85) of the patients reported a decrease in the size of the painful area after first treatment, while following re‐treatment, 44% (*n = *42) of patients reported a reduction at Week 2. At Week 12 following treatment and re‐treatment, a decrease in the size of the painful area from baseline and re‐treatment baseline was reported in 28% (*n = *99) and 35% (*n = *55) of patients, respectively.

### 3.7 Tolerability

Immediately after application, 157 patients (41%) presented with a mild application site reaction, 148 (39%) a moderate application site reaction and 43 (11%) a severe application site reaction. Thirty‐seven (10%) patients required rescue medication for application site reactions, while 201 patients (52%) required rescue treatment for treatment‐induced pain. During the week following treatment, the proportion of patients reporting moderate and severe application site reactions decreased to 7% (*n = *27). In addition, the maximum pain intensity (mean NPRS pain ‘right now’ score) due to capsaicin 8% patch treatment, at the group level, decreased from 5.7 (SD 2.2) at first treatment baseline (after application) to 4.7 (SD 2.6) at Week 1 (Fig. 1A).

Following re‐treatment application, 72 patients (40%) showed a mild application site reaction, 57 (32%) a moderate reaction and 21 patients (12%) a severe reaction. Rescue medication was required by 14 (8%) patients for application site reactions and by 87 patients (48%) for treatment‐induced pain. At Week 1 following re‐treatment, moderate and severe application site reactions were observed in only 6% (*n = *10) of patients and the maximum pain intensity (mean NPRS ‘pain right now’ score) due to capsaicin 8% patch treatment decreased from 5.0 (SD 2.6) to 3.6 (SD 2.6) at the group level (Fig. 1B).

### 3.8 Concomitant medication due to PNP

There was no change in the overall use of concomitant medication for PNP from first treatment baseline (47%, *n = *382) to Week 12 (49%, *n = *381) (Table 8). The most common form of additional medication at baseline was over‐the‐counter analgesics, which were used by 95 patients (25%) at Week 2 and by 131 patients (34%) at Week 12. There was also no notable changes in the overall use of concomitant medication due to PNP from re‐treatment baseline (48%, *n = *181) to Week 12 (52%, *n = *178).

**Table 8:** Use of concomitant pain medications due to PNP at first treatment and re‐treatment

Medication category, *n* (%)	First treatment (*n = *382)		Re‐treatment (*n = *181)	
	Treatment visit	Week 12	Treatment visit	Week 12
Light analgesics and antipyretics	113 (30)	131 (34)	59 (33)	59 (33)
NSAID non‐steroidal anti‐inflammatory drugs	45 (12)	56 (15)	30 (17)	31 (17)
Combination^a^	22 (6)	26 (7)	12 (7)	12 (7)
Opioids	11 (3)	18 (5)	9 (5)	10 (6)
Local anaesthetics	7 (2)	11 (3)	6 (3)	6 (3)
Antidepressants	10 (3)	14 (4)	5 (3)	7 (4)
Anticonvulsants	6 (2)	8 (2)	4 (2)	4 (2)
Other	18 (5)	22 (6)	8 (4)	11 (6)

a Fixed‐dose combination medication containing active agents from more than one pain medication subgroup.

## 4 Discussion

This study demonstrates that in Scandinavian clinical practice, the capsaicin 8% patch provided effective pain relief in a proportion of patients with PNP and was generally well tolerated. The overall group of patients had a significant reduction in NPRS ‘usual’ pain from first treatment baseline and re‐treatment baseline to Weeks 2 through 8 along with clear improvements in PGIC and HRQoL throughout the study. The majority of application site reactions diminished within a week of treatment, and over half of patients treated were willing to undergo re‐treatment.

Aligned with previous clinical trials using the same patch, a reduction in mean NPRS ‘usual’ pain was reported at Week 1 following treatment and was sustained to the final visit (Backonja et al., 2008; Simpson et al., 2008; Haanpaa et al., 2016). Overall, 28% and 31% of patients reported a ≥30% decrease in pain from first treatment baseline and re‐treatment baseline to Weeks 2 through 8, respectively, and significant reductions (−1.05 and −0.75, respectively) (*p* ˂ 0.001) in mean NPRS ‘usual’ pain were observed. In addition to a reduction in pain, patients also experienced a decrease in the size of the estimated painful area at the group level, with 28% of patients reporting a decrease over 12 weeks. Although improvements in pain intensity and the proportion of ≥30% responders after one treatment are lower than previously reported in other capsaicin 8% patch trials (−2.00 to −2.37 and 42% to 56%, respectively) (Backonja et al., 2008; Irving et al., 2011; Clifford et al., 2012; Haanpaa et al., 2016), there is a similar trend in the data. Also, the heterogeneous patient population from a diagnostic perspective may have included subgroups of patients less susceptible to respond to this type of treatment. For example, in another real‐world study of the capsaicin 8% patch that included seven European countries, patients with post‐operative and post‐traumatic neuropathic pain had less improvement in pain over 8 weeks compared with patients with other aetiologies including postherpetic neuralgia and neuropathic back pain (Mankowski et al., 2017). Direct comparisons to other studies are not possible due to differences in trial design (i.e. real‐world vs. controlled study, timing of study visits, patient population).

In addition to pain relief, the overall health status at the group level, as measured by PGIC, was also improved, with over 50% of patients reporting an improvement (slight, much, very much) at Week 1 following first treatment. This result was sustained over the 12 weeks and after re‐treatment with a higher proportion of patients reporting a ‘much or very much’ improvement. This may be due to the overall further reduction in pain intensity after re‐treatment. In addition, improvements in HRQoL were also observed early after treatment from first treatment baseline and re‐treatment baseline to Week 1. From first treatment baseline to Week 2, the change in EQ‐5D‐3L health score at the group level (0.148 utils) indicates a clinically meaningful improvement in quality of life (Walters and Brazier, 2005) with 52% of patients experiencing an improvement. Together, these results are in agreement with a European real‐world study of the capsaicin 8% patch where 63% of patients had an improvement in PGIC at Week 2, and the EQ‐5D‐3L health increased by 0.199 utils from baseline to Week 2 (Mankowski et al., 2017).

In general, the capsaicin 8% patch was well tolerated, and in most cases, adverse application site reactions diminished within a week after treatment. Interestingly, on treatment visits, reported mean NPRS ‘usual’ pain was higher than treatment‐related pain (NPRS ‘pain now’ score). The proportion of patients with severe application site reactions corresponded well with those needing rescue measures for the treatment‐related application site reactions. Although only a third of patients experienced ≥30% reduction in pain, over half of patients were willing to receive a second treatment with the patch, with only 3% of patients discontinuing treatment due to an adverse drug reaction. Over half of patients willing to undergo re‐treatment had their second capsaicin 8% patch application at their final visit (90 days post‐treatment). About three‐quarters of the patients who completed re‐treatment were willing to continue to a third treatment. These results suggest that despite application site reactions, a large number of patients were willing to undergo re‐treatment.

Strengths of the present study include a real‐world setting, inclusion of patients with different PNP aetiologies, assessment of re‐treatment with the capsaicin 8% patch and the assessment of patients’ willingness to undergo re‐treatment. As this was a pragmatic study, it was not designed to determine treatment efficacy. Other limitations included a lack of systematic collection of data for adverse effects and concomitant medications. In addition, the size of the painful area, assessed through rough estimations by patients, could have been assessed more precisely during study visits, and the patient's treatment satisfaction was not included in the protocol. Moreover, the patches may not have covered the entire painful area in some patients with polyneuropathy, and for patients who did not receive re‐treatment on the final visit after first treatment, it would have been more accurate to perform re‐treatment baseline assessments at the re‐treatment visit (before patch application).

In conclusion, capsaicin 8% patch treatment in routine clinical practice led to moderate, yet statistically significant, improvements in pain relief with approximately a third of patients experiencing ≥30% reduction in pain after first and second treatment and over half of patients willing to have further treatment. In addition, the proportion of patients reporting an improvement in their health status almost doubled with re‐treatment with a large number of patients reporting improvements in quality of life.

## Supporting information


**Table S1**. Clinic visit attendees.
