# Supplementary material for: Pain‐relieving effectiveness, quality of life and tolerability of repeated capsaicin 8% patch treatment of peripheral neuropathic pain in Scandinavian clinical practice
Source: Eur J Pain. 2018 Feb 1;22(5):941–50. doi: 10.1002/ejp.1180 (PMC5947653; doi:10.1002/ejp.1180)
Supplement: Supplementary file 1 — Table S1. Clinic visit attendees. [file EJP-22-941-s001.docx]

**Supplementary Content**


**Table 1** Clinic visit attendees

| Week | Overall | | Denmark | | Norway | | Sweden | |
| --- | --- | --- | --- | --- | --- | --- | --- | --- |
|  | n | Median, days  (min–max) | n | Median, days  (min–max) | n | Median, days  (min–max) | n | Median, days  (min–max) |
| First treatment |  |  |  |  |  |  |  |  |
| Baseline | 382 |  | 93 |  | 93 |  | 196 |  |
| 1 – visit 1 | 282 | 4 (2–27) | 85 | 4 (2–11) | 53 | 5 (2–27) | 144 | 3 (2–12) |
| 1 – visit 2 | 114 | 7 (2–34) | 29 | 7 (3–14) | 27 | 9 (5–34) | 58 | 6 (2–26) |
| 2 | 280 | 15 (5–64) | 58 | 17 (5–12) | 77 | 15 (7–41) | 145 | 15 (6–64) |
| 4 | 270 | 35 (7–74) | 68 | 35 (21–74) | 76 | 36 (14–56) | 126 | 34 (7–72) |
| 8 | 216 | 64 (41–113) | 49 | 66 (55–82) | 74 | 65 (49–89) | 93 | 63 (41–113) |
| 12 | 382 | 93 (17–164) | 93 | 97 (40–148) | 93 | 100 (59–164) | 196 | 91 (17–156) |
| Re-treatment |  |  |  |  |  |  |  |  |
| Baseline* | 181 |  | 42 |  | 47 |  | 92 |  |
| 1 – visit 1 | 135 | 4 (1–31) | 36 | 4 (2–7) | 37 | 6 (2–14) | 62 | 4 (1–31) |
| 1 – visit 2 | 38 | 11 (3–48) | 7 | 8 (3–12) | 16 | 16 (6–48) | 15 | 7 (4–36) |
| 2 | 108 | 16 (7–66) | 21 | 16 (8–34) | 36 | 18 (11–66) | 51 | 16 (7–52) |
| 4 | 103 | 35 (22–86) | 26 | 34 (23–51) | 36 | 41 (22–86) | 41 | 34 (23–67) |
| 8 | 82 | 64 (31–106) | 15 | 61 (43–79) | 32 | 67 (50–85) | 35 | 62 (31–106) |
| 12 | 181 | 93 (30–226) | 42 | 93 (38–226) | 47 | 99 (43–144) | 92 | 92 (30–196) |

*Baseline for re-treatment was the Week 12 assessment from first treatment.
